# Determination of thresholds for minimally important difference and clinically important response on the functional outcomes of sleep questionnaire short version in adults with narcolepsy or obstructive sleep apnea

**DOI:** 10.1007/s11325-020-02270-3

**Published:** 2021-01-04

**Authors:** Terri E. Weaver, Diane M. Menno, Morgan Bron, Ross D. Crosby, Susan Morris, Susan D. Mathias

**Affiliations:** 1 College of Nursing, Department of Biobehavioral Nursing Science and College of Medicine, Department of Medicine, Division of Pulmonary, Critical Care, Sleep & Allergy, University of Illinois at Chicago, 845 South Damen Avenue MC 802, Chicago, IL 60612 USA; 2 Jazz Pharmaceuticals, Philadelphia, PA USA; 3 Jazz Pharmaceuticals, Palo Alto, CA USA; 4 Sanford Center for Bio-Behavioral Research, Fargo, ND USA; 5 Health Outcomes Solutions, Winter Park, FL USA

**Keywords:** Quality of life, Treatment outcome, Clinically important difference, Clinical significance, Hypersomnolence disorders, Sleep-disordered breathing, 10.150 Sleep disordered breathing therapy, 20.400 Narcolepsy, 100.300 Daytime sleepiness

## Abstract

**Purpose:**

This study estimated thresholds for clinically important responses and minimally important differences for two indicators of improvement for the 10-item version of the functional outcomes of sleep questionnaire (FOSQ-10).

**Methods:**

Participants with excessive daytime sleepiness with narcolepsy or obstructive sleep apnea received 12 weeks of solriamfetol treatment. Participants completed the FOSQ-10 and other patient-reported outcome measures, including the single-item patient global impression of change (PGI-C) assessment. Clinicians completed the single-item clinician global impression of change (CGI-C) for each participant. Data from the two studies were analyzed separately, both without regard to treatment assignment. In total, 690 participants (47% female, mean age 48 years, 77% Caucasian, 91% from North America) were enrolled. Two clinically important changes, defined as a minimally important difference and a clinically important response, were determined using distribution and anchor-based analyses. A receiver operating characteristic analysis was used to determine the optimal FOSQ-10 change threshold.

**Results:**

Spearman correlations between change in FOSQ-10 scores and PGI-C and CGI-C were − 0.57 and − 0.49 for participants with narcolepsy and − 0.42 and − 0.37 for participants with obstructive sleep apnea. Receiver operating characteristic analysis suggested minimally important difference and clinically important response estimates of 1.7 and 2.5 and 1.8 and 2.2 points in narcolepsy and obstructive sleep apnea, respectively.

**Conclusions:**

Minimally important difference and clinically important response estimates for the FOSQ-10 for adults with excessive daytime sleepiness in narcolepsy or obstructive sleep apnea will be helpful for interpreting changes over time and defining a clinical responder.

**ClinicalTrials.gov identifiers:**

NCT02348593 (first submitted January 15, 2015) and NCT02348606 (first submitted January 15, 2015)

## Introduction

Excessive daytime sleepiness (EDS) can be caused by a variety of conditions, including narcolepsy, obesity, sleep apnea, and other chronic diseases [1]. EDS is a clinical hallmark of narcolepsy and obstructive sleep apnea (OSA) [2, 3]. Often the first symptom observed, EDS occurs in 100% of those with narcolepsy [2]. Among those with OSA, the prevalence of EDS has been shown to vary by gender [4] and OSA severity [5]. Among patients treated with continuous positive airway pressure (CPAP) therapy, residual EDS has been reported by 9 to 22%, depending on average duration of nightly CPAP use [6]. Those with EDS associated with OSA or narcolepsy experience fatigue, impaired work performance, emotional worry, decreased focus, impaired personal and family relationships, and falling asleep while driving [7, 8]. Additionally, EDS can impair functionality and may result in poorer social engagement [9].

The Functional Outcomes of Sleep Questionnaire (FOSQ) is a 30-item, disease-specific, quality-of-life questionnaire designed to assess the impact of EDS on activities of everyday living and the extent to which these activities are improved by effective treatment [9]. An abbreviated 10-item version of the questionnaire (FOSQ-10) was developed as an alternative for use in clinical trials and everyday clinical practice [10]. The reliability, validity, and responsiveness of the FOSQ-10 have been previously demonstrated [10], but estimates of how much change is clinically meaningful have not been previously established.

Establishing thresholds that characterize a meaningful change to patients can help clinicians interpret results from patient-reported outcome measures and may inform clinical decisions regarding patient care [11, 12]. These thresholds can also help researchers interpret the clinical significance of changes within a study and estimate the necessary sample size for randomized trials [11].

We sought to identify two thresholds for change for the FOSQ-10. The first, the minimally important difference (MID), is defined as the smallest change that a patient is able to identify. The second, the clinically important response (CIR), indicates a change that represents a relevant treatment benefit [13]. By establishing these thresholds, we intend to improve the understanding of how to interpret increments of change within the FOSQ-10.

## Methods

### Study population and data sources

Data were analyzed from participants with EDS associated with narcolepsy or OSA who were enrolled in one of two phase 3, multicenter, randomized, double-blind, 12-week studies [14, 15] of solriamfetol, a dopamine and norepinephrine reuptake inhibitor approved in the USA and European Union for these disorders. Full details of these studies’ methods, participant populations, and primary efficacy and safety outcomes have been reported previously [14, 15]. Both studies were approved by institutional review board or ethics committees at each site, and all participants provided written informed consent [14, 15]. Most inclusion and exclusion criteria were similar: both trials included adult participants (aged 18–75 years) with a body mass index from 18 to < 45 kg/m^2^ and examined the safety and efficacy of solriamfetol for EDS. However, some inclusion criteria differed slightly; one trial involved participants with OSA, while the other enrolled those with narcolepsy without moderate to severe OSA. Additionally, while inclusion criteria regarding the Epworth Sleepiness Scale (ESS) were the same in the two trials, there were slightly different requirements for baseline sleep latency based on the Maintenance of Wakefulness Test (MWT): patients in the OSA trial were included if their sleep latency was less than 30 min, whereas patients in the narcolepsy trial were included if their sleep latency was less than 25 min. For the OSA study, participants had OSA diagnosed according to *International Classification of Sleep Disorders, 3rd edition* (ICSD-3) criteria and current or prior use of a primary OSA therapy, including a CPAP machine, oral appliance, or surgical intervention [15]. Participants in the narcolepsy study were diagnosed according to ICSD-3 criteria or *Diagnostic and Statistical Manual of Mental Disorders, 5th edition* [14] criteria. Treatment groups were also slightly different in the two studies: while each trial included groups receiving 75 mg, 150 mg, and 300 mg (as well as a placebo group), the OSA trial also included an additional dose comparator of 37.5 mg that the narcolepsy trial did not.

Participants completed the MWT and ESS. The MWT is administered by trained clinicians and measures the ability to stay awake for a defined period of time, requiring participants to try to stay awake as long as possible while seated in a comfortable chair in a darkened room [16]. The ESS is a self-administered questionnaire that asks participants to rate their likelihood of dozing off or falling asleep during eight different activities [17]. Participants also completed the FOSQ-10 and the single-item Patient Global Impression of Change (PGI-C) rating. Clinicians completed a single-item Clinician Global Impression of Change (CGI-C) rating for each participant. Both impression of change ratings (PGI-C and CGI-C) assessed the change in the participant’s condition from baseline to week 12 using a 7-point scale ranging from 1 (very much improved) to 7 (very much worse). In contrast to the original 30-item FOSQ, the FOSQ-10, which was originally developed for application in the clinical setting, is an abbreviated 10-item version that can be readily used in clinical practice and research. The FOSQ-10 is scored to provide a total score containing items related to general productivity, activity level, vigilance, social outcomes, and intimacy. It has previously been shown to have strong psychometric properties and to perform similarly to the FOSQ-30, with high internal consistency, reliability, and effect sizes and pre- and post-treatment differences that are highly correlated with the 30-item version [10].

### Statistical analysis

Data from the two studies (NCT02348593 and NCT02348606) were analyzed separately, both without regard to randomization group. The analyses were based upon the modified intent-to-treat population that includes data from participants who were randomized, received at least one dose of study medication (solriamfetol or placebo), and have baseline and at least one post-baseline evaluation.

Demographic characteristics (including gender, age, country of residence, region, race, and ethnicity) were summarized descriptively, along with baseline values of the FOSQ-10 and ESS, using frequency and percent for categorical variables and mean, standard deviation (SD), and range for continuous variables.

Distribution-based analyses were used to estimate the minimal detectible change (MDC), which represents the smallest change that can be reliably distinguished from random fluctuation and thus represents the lower bound for estimates of clinically important change. The distribution-based measures that served as estimates of the MDC were 1.0 standard error of measurement (SEM), a value of 0.5 Cohen’s *d* or the standardized effect size [18], and a value of 0.5 Guyatt’s statistic (also referred to as the responsiveness statistic) [19].

Next, an anchor-based approach that incorporated either PGI-C or CGI-C (as assessed at week 12) as the anchor was used to determine what magnitude of change on the FOSQ-10 represented a meaningful change from the patient’s or clinician’s perspective. This type of approach has previously been used for interpreting changes that are meaningful on the ESS and other patient-reported outcomes [20, 21].

Spearman correlation coefficients were calculated between each of the anchors (i.e., the impression of change rating) and the change in FOSQ-10 score from baseline to week 12 or early termination. Correlations ≥ 0.30 in absolute value are recommended to demonstrate suitable anchors and to avoid contamination of interpretation thresholds with noise [22]. Participants were categorized into groups based on their CGI-C and PGI-C ratings, and the change in FOSQ-10 scores from baseline to week 12 or early termination was summarized by category and represented graphically using box plots. Since few participants chose “very much worse,” “much worse,” or “worse” on the PGI-C and CGI-C, these categories were combined for this analysis.

We evaluated two thresholds for determining a CIR. The first was a PGI-C or CGI-C rating of “minimally improved” or better, and the second was a rating of “much improved” or better. To characterize the association between each specific FOSQ-10 change score and this definition of a CIR, the sensitivity and specificity were calculated, and receiver operating characteristic (ROC) curves were derived using logistic regression analyses. ROC curves simultaneously describe the sensitivity and specificity of a predictive measure as different cutoff values are applied. The optimal values for the FOSQ-10 changes that were best associated with each of the PGI-C or CGI-C threshold values for clinically important change (based on equal importance of sensitivity and specificity) were generated from the corresponding ROC curves. The area under the ROC curve, reported as the C-statistic from the logistic regression model, represents the overall ability of model predictions to discriminate between individuals who do and do not experience clinically important change at the specified level.

## Results

### Patient characteristics

The analysis included a total of 690 participants across the two studies (Table 1). Among participants with narcolepsy (*N* = 231), 65% were female, 80% were white, and 81% were from North America, and the mean age was 36 years. The mean (SD) ESS score for these participants was 17.2 (3.18), and the mean (SD) FOSQ-10 score was 11.7 (3.03). Among participants with OSA (*N* = 459), 38% were female, 76% were white, and 97% were from North America, and the mean age was 54 years. OSA participants had a mean (SD) ESS score of 15.2 (3.32) and a mean (SD) FOSQ-10 score of 13.9 (3.01).

**Table 1:** Demographic and baseline characteristics

Characteristic	Study	
	Narcolepsy	Obstructive sleep apnea
Sample size (*N*)	231	459
Gender, *n* (%)		
Female	150 (64.9%)	172 (37.5%)
Male	81 (35.1%)	287 (62.5%)
Age, year		
Mean	36.20	53.86
SD	13.15	10.96
Range	18–70	20–75
Region, *n* (%)		
Europe	44 (19.0%)	15 (3.3%)
North America	187 (81.0%)	444 (96.7%)
Race, *n* (%)		
American Indian	2 (0.9%)	1 (0.2%)
Asian	6 (2.6%)	17 (3.7%)
Black	33 (14.3%)	87 (19.0%)
Multiple	5 (2.2%)	4 (0.9%)
Pacific Islander	1 (0.4%)	2 (0.4%)
White	184 (79.7%)	348 (75.8%)
Ethnicity, *n* (%)		
Hispanic	10 (4.3%)	40 (8.7%)
Not Hispanic	221 (95.7%)	419 (91.3%)
Mean (SD) FOSQ-10 score	11.7 (3.03)	13.9 (3.01)
Mean (SD) ESS score	17.2 (3.18)	15.2 (3.32)
Mean MWT sleep latency, min^a,b^	6.2–8.7^c^	12.1–13.6^c^

SD, standard deviation; FOSQ-10, Functional Outcomes of Sleep Questionnaire-10; ESS, Epworth Sleepiness Scale; MWT, Maintenance of Wakefulness Test; OSA, obstructive sleep apnea

^a^Inclusion criteria included baseline MWT sleep latency < 25 min for participants with narcolepsy and < 30 min for participants with OSA

^b^Narcolepsy, *n* = 227 (placebo, *n* = 57; combined solriamfetol, *n* = 170); OSA, *n* = 450 (placebo, *n* = 111; combined solriamfetol, *n* = 339)

^c^Range across all treatment groups

### Estimating the MDC

In participants with narcolepsy, the 1.0 SEM, 0.5 Cohen’s *d*, and 0.5 Guyatt’s statistic were 1.26, 1.49, and 1.03, respectively. In participants with OSA, the values were 1.48, 1.51, and 1.21, respectively.

### Descriptive analyses

**Fig. 1:**
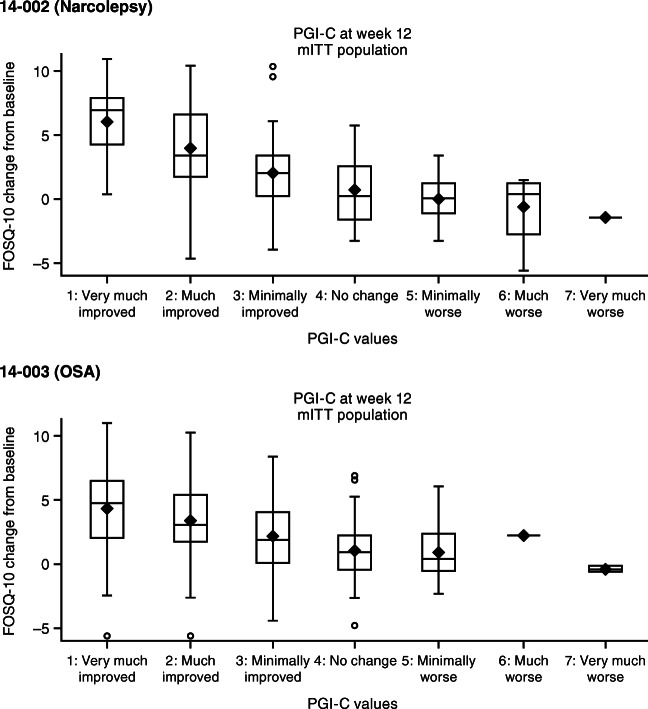
Change in FOSQ-10 by PGI-C rating. FOSQ-10, Functional Outcomes of Sleep Questionnaire-10; PGI-C, Patient Global Impression of Change; mITT, modified intent-to-treat; OSA, obstructive sleep apnea

Spearman correlations between each of the anchors, PGI-C and CGI-C, and changes in FOSQ-10 from baseline to week 12 or early termination were − 0.57 and − 0.49 for participants with narcolepsy and − 0.42 and − 0.37 for participants with OSA, confirming that both ratings were suitable for use in anchor-based MID analysis [23]. In general, mean FOSQ-10 change scores were higher in participants whose ratings of change (PGI-C or CGI-C) indicated greater improvement (Table 2 and Fig. 1).

**Table 2:** Mean changes in FOSQ-10 by CGI-C and PGI-C ratings

Study		Mean change in FOSQ-10 scores^a,b^				
		Very much improved	Much improved	Minimally improved	No change	Worse^c^
14–002 (Narcolepsy)	PGI-C	6.15 (2.98) [*n* = 17]	4.03 (2.95) [*n* = 55]	2.05 (2.76) [*n* = 56]	0.98 (2.42) [*n* = 41]	− 0.08 (2.05) [*n* = 22]
	CGI-C	4.43 (3.00) [*n* = 18]	4.28 (3.00) [*n* = 58]	1.74 (2.85) [*n* = 60]	0.85 (2.35) [*n* = 45]	0.80 (3.25) [*n* = 14]
14–003 (OSA)	PGI-C	4.32 (3.23) [*n* = 66]	3.32 (2.74) [*n* = 137]	2.08 (2.71) [*n* = 95]	0.97 (2.07) [*n* = 89]	0.81 (2.17) [*n* = 18]
	CGI-C	3.90 (3.35) [*n* = 81]	3.40 (2.67) [*n* = 128]	1.66 (2.44) [*n* = 91]	1.31 (2.41) [*n* = 97]	0.95 (2.59) [*n* = 8]

FOSQ-10, Functional Outcomes of Sleep Questionnaire-10; CGI-C, Clinician Global Impression of Change; PGI-C, Patient Global Impression of Change; OSA, obstructive sleep apnea

^a^Values in each cell are: mean (SD) [*n*] FOSQ-10 score

^b^FOSQ-10 scores range from 5 to 20, with higher scores representing better functioning. Larger change scores represent greater improvement

^c^Worse indicates minimally worse, much worse, and very much worse, combined due to small sample size

The mean changes in FOSQ-10 scores for those who rated themselves (PGI-C) or whose clinician rated them (CGI-C) as “minimally improved” were 2.05 and 1.74 for narcolepsy and 2.08 and 1.66 for OSA. These values exceed the highest estimated value for the MDC, approximately 1.5 in both indications. In participants who were rated as “much improved” on the PGI-C or CGI-C, the mean changes in FOSQ-10 were 4.03 and 4.28, respectively, for participants with narcolepsy and 3.32 and 3.40 for participants with OSA (Table 2).

### Estimating the MID and CIR

The results of the ROC analyses of the change in FOSQ-10 that was best associated with each definition of improvement (i.e., “minimally improved” or “much improved” or “very much improved” and “much improved” or “very much improved”) are presented in Table 3 as empirical estimates of the MID and CIR, respectively, in participants with narcolepsy and OSA. The areas under the ROC curves were also very similar.

**Table 3:** FOSQ-10 thresholds from ROC analyses

Study	Anchor	Level of change targeted	Change in FOSQ-10 scores	Sensitivity	Specificity	C-statistic
To estimate the clinically important response						
14–002 (Narcolepsy)	PGI-C	Much improved or better	2.50	0.681	0.689	0.789
	CGI-C	Much improved or better	2.50	0.697	0.697	0.773
14–003 (OSA)	PGI-C	Much improved or better	2.17	0.635	0.644	0.719
	CGI-C	Much improved or better	2.17	0.622	0.643	0.711
To estimate the minimally important difference						
14–002 (Narcolepsy)	PGI-C	Minimally improved or better	1.67	0.695	0.698	0.755
	CGI-C	Minimally improved or better	1.71	0.647	0.644	0.708
14–003 (OSA)	PGI-C	Minimally improved or better	1.83	0.681	0.682	0.724
	CGI-C	Minimally improved or better	1.88	0.623	0.638	0.672

FOSQ-10, Functional Outcomes of Sleep Questionnaire-10; ROC, receiver operating characteristic; PGI-C, Patient Global Impression of Change; CGI-C, Clinician Global Impression of Change; OSA, obstructive sleep apnea

**Fig. 2:**
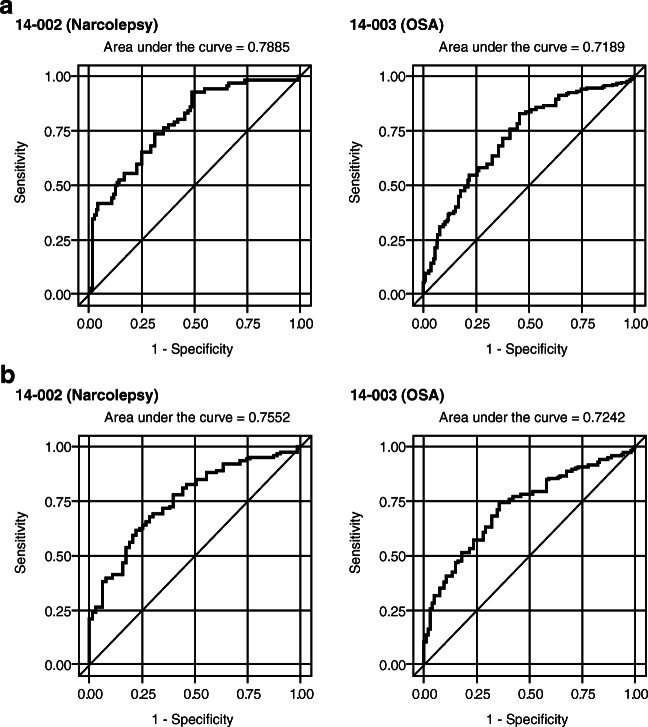
ROC curves for PGI-C response = (**a**) “much improved or better and (**b**) “minimally improved or better” ROC, receiver operating characteristic; PGI-C, Patient Global Impression of Change; OSA, obstructive sleep apnea

**Fig. 3:**
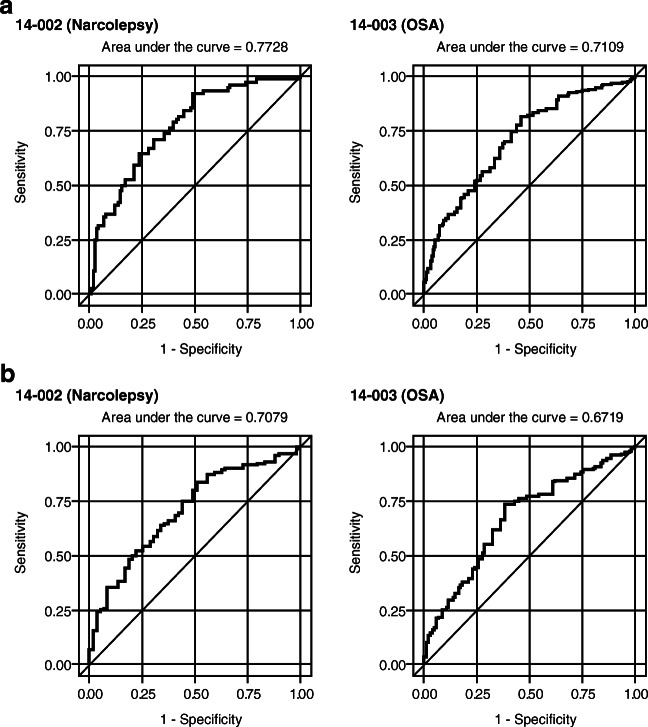
ROC curves for CGI-C response = (**a**) “much improved or better” and (**b**) “minimally improved or better” ROC, receiver operating characteristic; CGI-C, Clinician Global Impression of Change; OSA, obstructive sleep apnea

The C-statistics (areas under the ROC curve) for the analyses of FOSQ-10 changes associated with PGI-C or CGI-C ratings of “much improved” or “very much improved” were between 0.71 and 0.79, indicating good accuracy for predicting a true response (Fig. 2 and Fig. 3). For reference, a perfect classifier of responders would have a C-statistic of 1.0; a random classifier would have a value of 0.5 [24]. Sensitivity and specificity were moderate, with values between 0.62 and 0.70. The optimal FOSQ-10 changes corresponding to a CIR in participants with narcolepsy were slightly higher than in participants with OSA, 2.5 compared to 2.2. Values obtained with the two anchors were the same, indicating concordance between participants’ and clinicians’ ratings of change at this level.

In the analyses of FOSQ-10 changes associated with PGI-C or CGI-C ratings of “minimally improved,” “much improved,” or “very much improved,” the C-statistics were between 0.67 and 0.76 (Fig. 2 and Fig. 3). Sensitivity and specificity were again moderate, with values between 0.62 and 0.70. The optimal FOSQ-10 changes corresponding to minimal improvement in participants with narcolepsy and OSA were more similar, with values of 1.7 for both anchors in narcolepsy and values of 1.8 and 1.9 for PGI-C and CGI-C in participants with OSA.

## Discussion

This aim of this study was to provide guidance on how to interpret within-patient changes in the FOSQ-10 over time by estimating thresholds for the MID and CIR. Multiple approaches were employed to provide an estimate of two levels of patient-perceived change in the FOSQ-10 total score: that of minimal change (MID) and the CIR. This work provides clinicians and researchers with benchmarks for evaluating and interpreting FOSQ-10 results in a manner that associates the patient or clinician perception of improvement with the FOSQ-10 total score. To ensure a robust estimate, we used multiple anchors and integrated results from both anchor-based and distribution-based estimates, as well as ROC analyses, in accordance with established recommendations [22].

This study also provided the opportunity to compare levels of change in the FOSQ-10 in participants with narcolepsy and OSA. The MID values for narcolepsy and OSA participants were 1.7 and 1.8, respectively, and the CIR values for narcolepsy and OSA participants were 2.5 and 2.2, respectively.

There is increasing recognition that the clinical significance of a change cannot be inferred from statistical significance alone. Determining clinical significance requires an understanding of how the change is perceived and experienced by the patient. The estimation of the MID and CIR provides clinicians and researchers with benchmarks for two levels of change, as perceived by patients, specifically the smallest change that a patient can identify (MID) and that which represents a relevant treatment benefit (CIR). Having both benchmarks available provides the opportunity to evaluate the levels of change of FOSQ-10 scores that may occur over time. The use of many of the anchor-based and distribution-based methods employed in the current study traces back to the early 1990s [23], and these methods have been used individually or together to estimate meaningful change in a variety of diseases, including cancer [25], heart disease [26], chronic obstructive pulmonary disease [27], urinary incontinence [25], inflammatory bowel disease [28], arthritis [29], and asthma [30]. The use of ROC analysis to empirically determine cutoff values has been utilized in previous studies to establish a CIR [13], including in studies of narcolepsy using the PGI-C or CGI-C as anchors [20, 21].

These analyses have several notable strengths. First, the analyses incorporated both distribution- and anchor-based methods. Two anchors, one based on a PGI-C and the other on a CGI-C, were used. Notably, the anchors reflected similar mean changes in FOSQ-10 scores, thereby strengthening the interpretation of what changes in FOSQ-10 scores represent. Additionally, the estimation of the MID and CIR was derived in two distinct patient populations with EDS. However, a limitation is that few participants in the current study experienced a worsening in their condition, precluding the estimation of either an MID or a CIR for worsening symptoms. This is consistent with results previously reported from these studies that indicated improvements in EDS with solriamfetol treatment, as demonstrated by least square mean decreases in ESS scores (narcolepsy, − 3.8 to − 6.4; OSA, − 5.0 to − 7.9) and increases in mean MWT sleep latency (narcolepsy, + 4.7 to + 12.3 min; OSA, + 4.7 to + 13.0 min) from baseline to week 12 relative to placebo (ESS scores: narcolepsy, − 1.6; OSA, − 3.3; MWT sleep latency: narcolepsy, 2.1 min; OSA, 0.2 min) [14, 15]. This is also consistent with recently published data from the same studies used in this analysis that demonstrated moderate to high correlations between changes in FOSQ-10 and ESS scores or mean MWT sleep latency [31]. Additionally, we note that the data presented here were obtained in the context of double-blind, placebo-controlled, randomized, parallel-group, phase 3 studies evaluating the effects of solriamfetol in the treatment of EDS. It is unclear to what extent the current findings are generalizable to other patient populations with EDS or with other therapies.

Despite these limitations, the CIR estimate for the FOSQ-10 overall score of 2.2 to 2.5 for adult populations with OSA or narcolepsy treated for EDS will be valuable for interpreting changes over time and defining a clinical responder. Future research in other populations or those receiving other therapies for EDS will add to this work.

## Data Availability

All relevant data are provided within the manuscript and supporting files.
